# Predicting Pro-environmental Intention and Behavior Based on Justice Sensitivity, Moral Disengagement, and Moral Emotions – Results of Two Quota-Sampling Surveys

**DOI:** 10.3389/fpsyg.2022.914366

**Published:** 2022-06-24

**Authors:** Susanne Nicolai, Philipp Franikowski, Susanne Stoll-Kleemann

**Affiliations:** ^1^Department of Sustainability Science and Applied Geography, Institute of Geography and Geology, University of Greifswald, Greifswald, Germany; ^2^Department of Personality Psychology and Psychological Assessment, Institute of Psychology, University of Greifswald, Greifswald, Germany

**Keywords:** justice sensitivity, climate justice, moral disengagement, moral emotions, pro-environmental behavior, pro-environmental intention, behavior change

## Abstract

The effects of climate change lead to increasing social injustice and hence justice is intrinsically linked to a socio-ecological transformation. In this study, we investigate whether justice sensitivity motivates pro-environmental intention (PEI) and behavior (PEB) and, if so, to what extent emotions and moral disengagement determine this process. For this purpose, we conducted two quota-sampling surveys (Study 1: *N* = 174, Study 2: *N* = 880). Multiple regression analyses in both studies suggest that a higher perception of injustice from a perpetrator’s, beneficiary’s, and observer’s perspective is associated with an increased PEI. However, moral disengagement best predicted PEB and PEI. Guilt and authentic pride were found to be emotional predictors of PEI. Additionally, mediation analyses demonstrated that guilt mediates the connection between both perpetrator and beneficiary sensitivity and PEI. These results suggest that when the predominant originators of climate change (i.e., individuals from industrialized countries) perceive global climate injustice from the perspective of a beneficiary or a perpetrator, they experience guilt and have a higher PEI. Based on this mechanism, it seems promising to render global injustice more salient to those responsible for activities that lead to climate change to motivate them to adapt their behavior. The role of moral disengagement and victim sensitivity as barriers to pro-environmental behavior is discussed in this context.

## Introduction

The latest report by the Intergovernmental Panel on Climate Change [IPCC] (2022) suggests that it is not only natural conditions that determine who is most affected by the climate crisis, but rather the living conditions of the individuals. Limited economic resources, suffering from inequality, enduring political instabilities and wars, or limited access to basic resources such as clean water are only a few examples of disadvantages faced by individuals due to climate change. Ultimately, human rights, such as the right to life, health, and subsistence are endangered by the consequences of climate-damaging behavior, i.e., behavior that emits a lot of greenhouse gases and, therefore, forces climate change. Due to unequally distributed resources (e.g., money, political power), climate change disproportionally affects the Global South, younger generations, women, and people with low income more intensely than the Global North, older generations, men, and people with high income (Intergovernmental Panel on Climate Change [IPCC], 2022). In all of these cases, the same disproportion can be found, namely that the individuals who are least responsible for climate change are more affected by its consequences than those most responsible for it. For example, from 1990 to 2015, the global wealthiest 10% were responsible for 46% of emission growth whereas the global poorest 50% were responsible for only 6% of the total emission growth (Kartha et al., 2020). As a consequence, discussions concerning climate change comprise ethical and moral issues regarding justice (Caney, 2010; Boom et al., 2016; De Smet et al., 2016; Alves and Mariano, 2018). Therefore, the term *climate justice* incorporates various justice principles in the domain of climate change. These include *distributive justice*, which considers the allocation of costs and benefits among people, *procedural justice*, which points out who can participate in decision-making processes, and *recognition*, which involves respectful and appropriate “consideration of diverse cultures and perspectives” (Intergovernmental Panel on Climate Change [IPCC], 2022, p. 9).

Justice was found to be a fundamental human motive (Montada, 2007; Baumert et al., 2013a). Therefore, we hypothesized that justice is not only the target but also a cause of pro-environmental intention, and, in turn, a motivator of pro-environmental behavior. Hence, we investigated whether justice sensitivity motivates individuals to behave (more) pro-environmentally. In this article, we apply findings from justice sensitivity research to the context of the current climate crisis and investigate the predictive power of justice sensitivity toward the intention of a pro-environmental behavioral change. Moreover, we investigate the associations of pro-environmental behavior change with moral emotions and moral disengagement.

In the context of climate justice, people in industrialized countries can be regarded as the beneficiaries of the detrimental living conditions of people from less developed countries, since the standard of living in industrialized countries is directly connected to the exploitation of natural and human resources in less developed countries (Dorninger et al., 2021; Hickel et al., 2022). However, many people may not be completely aware of this fact as the actions that result in driving climate change are probably perceived as an “unintentional, if unfortunate, side effect” of goal-directed behavior (Markowitz and Shariff, 2012). Markowitz and Shariff (2012) call this phenomenon the *blamelessness of unintentional action*. Unintentionally caused consequences are judged less harshly than equally severe but intentionally caused consequences (Guglielmo et al., 2009). However, the existence or lack of an intention does not change the consequences of an action. Therefore, we hypothesize that people need to identify themselves as responsible to renounce their privileges and act in solidarity with the disadvantaged. Moreover, we assume that this perception is a motivator for pro-environmental behavior change and, therefore, is pivotal to achieving climate justice. Consequently, we focus on justice sensitivity in this article. In the following, we contextualize the role of justice sensitivity on pro-environmental behavior and intention by investigating the joint prediction of pro-environmental behavior and intention with justice sensitivity, emotions, and moral disengagement.

Justice was found to be a fundamental human motive (Montada, 2007; Baumert et al., 2013a). That is, individuals generally seek to establish justice while avoiding injustice (Lerner, 1977), and hence individuals want themselves and others to be treated fairly and are willing to behave according to justice principles (Baumert et al., 2013b). To establish justice, individuals are willing to give up their own advantages (Engel, 2011), act sustainably (Kals and Becker, 2006), or reject a beneficial but unfair deal (Sutter et al., 2020). A meta-analysis based on 182 studies on collective behavior confirmed that the more strongly individuals perceive a situation to be unjust, the more likely they are to participate in collective action (such as protest behavior) to improve the situation (van Zomeren et al., 2008). Baumert et al. (2013b) summarize:

Assuming that justice is a fundamental motive for individuals means that the perception of a potential injustice triggers emotional reactions (e.g., anger, moral outrage, compassion, guilt) and urges the individual to act in order to restore justice or to avoid the injustice. Hence, the concept of a human justice motive implies the assumption of a psychological link between the perception of (potential) injustice and affective and behavioral reactions (p. 161).

Although justice is a fundamental motive, individuals differ in their judgments concerning whether a situation (e.g., the climate crisis) is just or unjust. These differing assessments were found to be due to different principles of justice that vary across different individuals, contexts, situations, and relationships (Deutsch, 1985; Jasso et al., 2016; Sabbagh and Schmitt, 2016; Skitka et al., 2016). In this article, we focus on the first aspect, namely individuals. Dispositional interindividual differences were found to influence the perception of justice, i.e., differences in *justice sensitivity* (Lerner, 1977; Münscher, 2017; Preiser and Beierlein, 2017). Justice sensitivity is regarded as a personality trait (Schmitt et al., 1995), as it was found to be consistent across situations and contexts (Schmitt et al., 2009). Justice sensitivity is defined as the tendency to perceive injustice that results in negative emotional responses. Together, the perception of injustice and its negative evaluation form action tendencies to mitigate the injustice (Baumert and Schmitt, 2016). This process is in line with the appraisal theory developed by Arnold (1960), which states that emotions, i.e., action tendencies, are the result of two cognitive processes: the factual cognition (here: Is the situation just or not?) and the evaluative cognition (here: evaluating injustice as negative – or positive).

Individuals react with different intensity to injustices they have suffered themselves (as victims), to injustices they perceive in everyday situations (as observers), to injustices from which they passively benefit (as beneficiaries), and to injustices they have committed themselves (as perpetrators; Montada and Maes, 2016). Although justice sensitivity is inherent to all four perspectives, they differ in several ways. On the one hand, a high justice sensitivity as an observer, beneficiary, or perpetrator reflects the desire for justice for others and a feeling of social responsibility. On the other hand, a high score in justice sensitivity as a victim reflects a desire for justice for oneself (Preiser and Beierlein, 2017). More precisely, individuals who score highly in perpetrator or beneficiary sensitivity rather experience prosocial concerns (e.g., existential guilt, social responsibility, or solidarity with the disadvantaged; Gollwitzer et al., 2005). Observer sensitives were found to feel empathy and, as a result, take responsibility (Schmitt et al., 2005). Those who perceive injustice from the perspective of the beneficiary, observer, or perpetrator are more likely to feel responsible (Ehrhardt-Madapathi et al., 2018). A greater sense of responsibility, in turn, is a promising predictor to engage in pro-environmental behavior (Kollmuss and Agyeman, 2002). As already indicated, victim sensitivity relates differently and in part contrarily to the other three perspectives. In terms of behavioral outcomes, victim sensitivity was positively related to delinquent behavior (e.g., fare or tax evasion; Preiser and Beierlein, 2017). Victim sensitives are afraid of being disadvantaged and behave in anticipation of such an injustice. Therefore, they easily abandon their moral standards (Gollwitzer et al., 2009; Schmitt et al., 2009; Münscher, 2017; Preiser and Beierlein, 2017), are rather jealous, neurotic, socially mistrusting, and paranoid (Schmitt et al., 2005) and fear the exploitation of their investments (Rothmund et al., 2014). To summarize, we can distinguish three pro-social sensitivities (perpetrator, beneficiary, and observer) from one pro-self sensitivity (victim). We assume that the three pro-social sensitivities are positively associated whereas the pro-self sensitivity is negatively associated with pro-environmental intention and behavior.

These associations are mirrored in emotional dispositions as individuals with a higher score in beneficiary or perpetrator sensitivity are more prone to experience guilt than those with a lower score. In contrast, individuals with a higher score in observer sensitivity tend to more easily be outraged than those with a lower score. Finally, individuals with a higher score in the area of victim sensitivity are inclined to feel more anger than those with a lower score (Preiser and Beierlein, 2017).

Emotions – especially, moral emotions – affect environmentally relevant behavior (Kals and Maes, 2002; Hahnel and Brosch, 2018; Harré, 2018; Landmann, 2020), and, therefore, are of particular interest in the area of climate injustice. We refer to *moral emotions*, as they “inhibit socially undesirable behavior and foster moral conduct” (Tangney, 1995). For example, in a study by Roeser (2012), in climate change communication, emotions promoted a better understanding of the moral meaning of climate change in comparison to neutral communication. As a consequence, emotions are a reliable motivator for pro-environmental behavior (Roeser, 2012). Corral-Verdugo (2013) even found that emotions have a higher impact on pro-environmental behavior than intentions. More precisely, emotions and intentions together explained more than half of the variance in pro-environmental behavior. In addition, emotions play a vital role in initiating behavioral *change* (Stoll-Kleemann et al., 2022a). Kollmuss and Agyeman (2002) state in their behavior change model that “the stronger a person’s emotional reaction, the more likely that person will engage in a new behavior.”

When speaking about the influence of emotions on pro-environmental behavior, we can distinguish between (1) emotions that are currently experienced, (2) emotions that people believe their actions will elicit (anticipated emotions), and (3) emotions that are generalized due to multiple repetitions. Venhoeven et al. (2013) also address the emotions that pro-environmental behavior elicits, although their findings are not relevant to this study. The three aforementioned emotion groups cause different effects. First, experimentally induced current emotions, e.g., by confronting participants with environmental damages to elicit guilt (Reese and Jacob, 2015), affected the pro-environmental intention or behavior of these individuals. However, these effects were found to be rather small, vary individually, and not remain stable over a period of time (Landmann, 2020, p. 10). Second, anticipated emotions can motivate people to engage in pro-environmental behavior as they either believe it will make them experience pleasure (Rezvani et al., 2017) or prevent them from experiencing displeasure (Onwezen et al., 2013). Third, according to the model of affect generalization (Paolini et al., 2016), emotional episodes can generalize when experienced sufficiently often or with high intensity. In the context of pro-environmental behavior, Landmann (2020) states that emotions only affect behavioral intentions if they are generalized. In line with the model of affect generalization, the emotions used in this work are not used as an emotional episode (currently experienced or anticipated), but rather as an affective attitude, that is, the proneness to feel these emotions.

In this study, we investigate the role of the moral emotions (1) pride, (2) gratitude, (3) guilt, and (4) shame. Considering the seven categories of environmentally relevant emotions by Landmann (2020), pride is one of the *self-praising emotions* which occur as a result of positive norm deviations. Gratitude belongs to *other-praising emotions* which are triggered by observing positive norm deviations in others. Guilt and shame cover *self-condemning emotions* that arise due to individual violations of personal norms. These different emotion types lead to different action tendencies. Whereas self-praising emotions reinforce one’s own positive behavior, e.g., through in-group favoring pro-environmental intentions, other-praising emotions lead to support for the source of these emotions, e.g., protecting nature. Self-condemning emotions motivate people to correct their behavior, e.g., by repairing environmental damage (Landmann, 2020).

Rezvani et al. (2017) found that when people anticipated feeling *pride* due to their pro-environmental behavior, they had a higher pro-environmental intention than individuals without this anticipation. Additional to this direct effect, anticipated pride also mediated the effect of personal moral norms on pro-environmental intentions. Experienced pride in environmental behavior increased an individual’s engagement in pro-environmental behavior (Bissing-Olson et al., 2016). However, two types of pride can and should be distinguished: *authentic* and *hubristic pride*. Authentic pride, on the one hand, focuses on the “self-in-action,” e.g., pro-environmental behavior, and is therefore tied to specific situations. Hubristic pride, on the other hand, focuses on the “self-as-actor” and generalizes across situations. This difference impacts behavior: whereas authentic pride positively predicted moral behavior, hubristic pride was negatively related to it (Krettenauer and Casey, 2015).

*Gratitude* is benefit-triggered and can be considered a trait and therefore may serve as a second positive emotion. Tam (2022) found that gratitude correlated with pro-environmental intention and behavior. However, manipulations of gratitude toward nature did not have robust effects on pro-environmental behavior change.

*Guilt* and *shame* due to environmental damage were consistently found to influence pro-environmental intention and behavior (Carrus et al., 2008; Ferguson and Branscombe, 2010; Gausel and Brown, 2012; Harth et al., 2013; Rees et al., 2014). When individuals are highly perpetrator- or beneficiary sensitive, they are also prone to feel guilty (Montada et al., 1986). Guilt results from regretting a specific behavior afterward but does not affect one’s self-identity. Shame, however, affects one’s self-identity as it goes along with a feeling of worthlessness and powerlessness (Tangney, 1995). Therefore, guilt tends to be tied to a specific behavior (similar to authentic pride), whereas shame is linked to self-identity (similar to hubristic pride). Rees et al. (2014) found that both emotions predicted positive behavioral intentions, but it was shame and not guilt that transformed the intention into actual behavior. However, Carrus et al. (2008) showed that anticipated guilt strongly affected an individual’s desire to use public transportation and engage in household recycling. The relationship between negative anticipated emotions and the pro-environmental intention was even stronger than that between attitudes and pro-environmental intention. Most research on emotions in pro-environmental behavior focused on guilt and pride. In direct comparison, pride and guilt were both significantly related to pro-environmental behavior and intention to engage in pro-environmental behavior. Shipley and van Riper (2022) found that anticipated pride and guilt had equal effects on pro-environmental behavior, but that experienced guilt had stronger effects on pro-environmental behavior than experienced pride. However, negative emotions must be dealt with consciously, as they result in aversive action tendencies. This is evident in the case of cognitive dissonance (Festinger, 1957). According to the theory of cognitive dissonance, emotional stress occurs when actions and beliefs are inconsistent. This emotional stress is highly uncomfortable and needs to be resolved. Based on Festinger’s theory, Bandura (2007, 2016) developed the concept of *moral disengagement*. Moral disengagement can be seen as a cognitive restructuring of a situation to justify inaction or subjectively wrong action (Bandura et al., 1996; Bandura, 2007, 2016) and does not only occur in individuals, but also in groups of individuals when harmful behavior is collectively morally justified (Bandura, 2002). These justification mechanisms include denial and diffusion of responsibility. Euphemistic labeling, advantageous comparison, in addition to minimizing, ignoring, or misconstruing the consequences are also forms of moral disengagement. Engaging in these strategies can be prevented by a high degree of moral self-regulation, that allows individuals to remain consistent with their standards of justice. Such a higher moral self-regulation and, hence, less moral disengagement, was found in perpetrator and beneficiary sensitive individuals in a longitudinal study by Maltese and Baumert (2016). Bandura (2007) already theorized how moral disengagement selectively contributes to increasing environmental damage. These mechanisms prevent individuals from engaging in pro-environmental behavior, even if they have already recognized the injustice of their behavior. Some of the mechanisms were investigated in a qualitative study (Stoll-Kleemann and O’Riordan, 2020) while the association of moral disengagement with high carbon behavior was found in a quantitative study (Stoll-Kleemann et al., 2022b). It is assumed that moral disengagement in high carbon behavior correlates positively with victim sensitivity, as both tendencies are self-serving and prevent an individual from taking responsibility for the environment. Therefore, moral disengagement is also assumed to correlate negatively with pro-environmental intention and behavior.

As argued above, pro-environmental behavior change is necessary to achieve climate justice. We hypothesize that climate justice is not only the ultimate goal of pro-environmental behavior but also its cause as justice functions as a motivator of behavior. To our knowledge, justice sensitivity perspectives have not yet been associated with pro-environmental intention and behavior. Based on the reported findings, the aim of this study is to investigate the extent to which perceptions of injustice predict pro-environmental intention and, in turn, might even predict pro-environmental behavior. More precisely, we assume that the pro-social justice sensitivities (perpetrator, observer, and beneficiary) are positively associated with pro-environmental intention (H1a). The pro-self justice sensitivity (victim) and the tendency to morally disengage are assumed to be negatively associated with pro-environmental intention (H2a). Furthermore, we assume an influence of moral emotions (guilt, shame, authentic and hubristic pride, and gratitude) on pro-environmental intention (H3b).

We also checked whether the three hypotheses also apply for pro-environmental behavior (H1b, H2b, and H3b). However, one must note that pro-environmental intentions might not be implemented in pro-environmental behavior and therefore may not account for all of the variance in pro-environmental behavior. This issue is known as the intention-behavior gap (Sheeran, 2002). If the predictions of Hypotheses 1–3 apply for pro-environmental intention, they might not necessarily apply to pro-environmental behavior.

**FIGURE 1:**
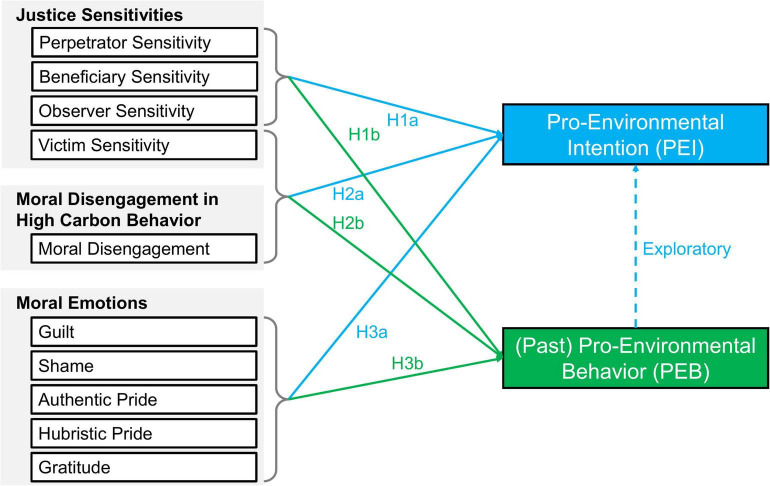
Hypotheses 1–3 (a and b) of the current Study.

As guilt results from both high perpetrator and beneficiary sensitivity, and the action tendency of guilt is to correct one’s behavior, we assume that guilt mediates the relationship between beneficiary (H4a) and perpetrator (H4b) sensitivity and pro-environmental intention. Moreover, we hypothesize an association between moral disengagement and victim sensitivity (H5). Hypotheses 1-3 can be found in Figure 1.

Study 1 was conducted to address Hypotheses 1 (a and b), 2 (a and b), and 5. Albeit the fact that pro-environmental intention cannot be regarded as a promising predictor for (future) pro-environmental behavior (Sheeran, 2002), past pro-environmental behavior was found to be a strong predictor for pro-environmental intentions (e.g., Bamberg et al., 2003; Carrus et al., 2008; Manca et al., 2020). As pro-environmental behavior was assessed retrospectively (as “past pro-environmental behavior”) in Study 1, we also checked if this finding can be replicated with our data (see Figure 1).

Study 2 addressed Hypotheses 3 (a and b) and 4 (a and b) and aimed to replicate the findings of Study 1 with a larger sample and a potentially more precise measure of (current) pro-environmental behavior, i.e., the “carbon footprint.”

## Study 1

### Materials and Methods

#### Participants

We recruited 220 German panelists on *meinungsplatz.de*. *Meinungsplatz.de* is a German survey platform offering a pool of 250,000 active participants from all social strata in Germany and Switzerland for market and opinion research purposes. All participants were compensated for their expenses following the policy of the panel service.

Quotas were defined concerning age and gender to ensure representativeness regarding these socio-demographic dimensions. The scheduled age and gender proportions were based on the Federal Statistical Office Germany (2019). Three panelists responded that they were 16 or 17 years old. We initially planned to only include data from participants that were at least 18 years old as these were requested from the panel provider. However, we decided to retain the data from these participants for the analyses as their participation is in accordance with the German legislation and we did not expect their response pattern to differ substantially from the participants that were at least 18 years old. The gender distribution in Germany is nearly equal (50.7% female, 49.3% male; Federal Statistical Office Germany, 2019) and therefore was assumed to be 50:50. Due to an unknown distribution of individuals who identify themselves as non-binary, these were not included (see Table 1).

**TABLE 1:** Proportions of German age groups (in 2018) and the derived sampling goals along with the final sample proportions in Study 1.

			Sample after exclusion		
Age group	Proportion	Quota sample before exclusion	Female	Male	Total
18–25 years	12%	28	10	5	15
26–35 years	15%	34	17	12	29
36–45 years	14%	30	12	12	24
46–55 years	18%	40	16	16	32
56–65 years	16%	36	14	15	29
66 years or older	24%	52	22	23	45

*Proportions are based on Federal Statistical Office Germany (2019). We assumed gender to be equally distributed (50:50).*

As exclusion criteria, we consulted the relative speed index (RSI; Leiner, 2019) and a social desirability score (Satow, 2012). The RSI is computed by dividing the median page completion time of the sample by the page completion time of the respective individual. A relative speed index of 2 indicates that an individual was twice as fast as the median of the total respondents. According to the recommendation of Leiner (2019), we removed 46 panelists with RSI > 2 as we assumed them to not have worked conscientiously. Additionally, we excluded 12 participants due to socially desirable response tendencies as they scored 7 or higher on the social desirability scale (see Instruments; Satow, 2012).

Therefore, the final sample for Study 1 comprised 174 participants: 91 females and 83 males (age *M* = 49.9, *SD* = 17.5, range = 16–80 years). Two χ^2^-tests for goodness of fit for age groups and gender, respectively, found no significant deviation from the proportions set for the recruiting procedure, either for gender, χ^2^(1) = 0.37, *p* = 0.544 or for age group, χ^2^(5) = 2.67, *p* = 0.751. The resulting sample therefore still represents the distribution of age and gender in the German population as a whole.

Sensitivity analyses using G*Power (Faul et al., 2007) indicated that with the given sample size of *N* = 174, α = 0.05, and a power of 1 – β = 0.80, in a linear multiple regression with five predictors (four justice sensitivities, and moral disengagement in high carbon behavior), small effect sizes of *R*^2^ = 0.071 and with a power of 1 – β = 0.95 small effect sizes of *R*^2^ = 0.105 could be detected.

#### Instruments

To test our hypotheses, we measured two dependent variables, pro-environmental behavior, and pro-environmental intention. The independent variables were the justice sensitivity perspectives and moral disengagement in high carbon behavior. The descriptive statistics and internal consistency measures of all variables of Study 1 can be found in Table 2.

**TABLE 2:** Descriptive statistics and internal consistency of all measures in Study 1.

Variable	*M*	*SD*	Skewness	Cronbach’s α
**Dependent variables**				
Past pro-environmental behavior	0.56	0.83	–0.20	0.41
Pro-environmental intention	4.26	0.18	–0.45	0.67
***Independent variables***				
Perpetrator sensitivity	2.99	1.34	0.13	0.82
Beneficiary sensitivity	4.97	1.10	–1.11	0.79
Observer sensitivity	4.21	1.17	–0.25	0.68
Victim sensitivity	3.30	1.47	0.14	0.81
Moral disengagement	2.18	1.01	1.14	0.96

##### Past Pro-environmental Behavior

To measure the first dependent variable, 10 pro-environmental behaviors were queried for the last year (i.e., 2019 as the survey was conducted in 2020). Due to the pandemic state of emergency during the survey period, we did not ask about the current behavior as this may have been affected by the COVID-19 pandemic. Therefore, we measured past pro-environmental behavior in Study 1. The participants were asked to indicate whether they pursued the specified behavior (“yes,” “no,” or “do not know”). To prevent an acquiescence bias (the tendency to answer with “yes” regardless of the question), some items were framed pro-environmentally and others from an environmentally damaging perspective. An example of an item is “In 2019, I purchased green electricity.” Based on the idea of the Campbell Paradigm (Kaiser et al., 2013), we addressed behaviors of varying “difficulty.” For example, we expected it to be more difficult to use green electricity (because it is more expensive) than to lower the washing temperature. The inverted items were subsequently reversed, and the relative frequency of pro-environmental behavior was computed for each person. The internal consistency of the scale was Cronbach’s α = 0.41. As the measure can be regarded as a formative measure (the latent construct is formed by the items) instead of a reflective measure (the latent construct is causal for the item responses), internal consistency estimates for reliability coefficients should not be interpreted and not considered for item selection (e.g., Bollen and Lennox, 1991; Rossiter, 2002). The scale can be found in Appendix A.

##### Pro-environmental Intention

To measure the second dependent variable, the same 10 items from the past pro-environmental behavior scale were re-formulated as intentions for the following year (i.e., 2021). For example, one item taken from the section above reads: “In 2021, I would like to (continue to) purchase green electricity.” As we asked for behavior intentions rather than actual behavior, answers were given on 6-point scales that expressed the participant’s approval of the items (ranging from “totally disagree” to “totally agree”). The internal consistency of the scale was Cronbach’s α = 0.67. The questionnaire can be found in Appendix B.

##### Justice Sensitivity (USS-8)

The injustice sensitivity scales (USS-8) of Beierlein et al. (2012) measure the four perspectives of justice sensitivity (victim, perpetrator, beneficiary, and observer). Each perspective is measured by two items, answered on a 6-point scale (ranging from “does not apply at all” to “fully applies”). An item example of beneficiary sensitivity is: “I feel guilty when I am undeservedly better off than others.” The reliabilities of perpetrator (Cronbach’s α = 0.82), beneficiary (Cronbach’s α = 0.89), and victim sensitivity (Cronbach’s α = 0.81) were good or even excellent; however, the reliability of observer sensitivity was questionable (Cronbach’s α = 0.68).

##### Moral Disengagement in High Carbon Behavior Scale

For Study 1, based on the questionnaire of Moore et al. (2012) on moral disengagement in general, we developed a German questionnaire that measures an individual’s propensity to morally disengage in the context of high carbon behavior (MD-HCB; Stoll-Kleemann et al., 2022b). The MD-HCB covers nine mechanisms of moral disengagement following Markowitz and Shariff (2012) and Bandura (2016) with two questions each (18 items in total). An exemplary item is “In terms of my carbon emissions, I don’t want to give much consideration to people who live very far away and whom I will never meet.” The reliability was excellent, with Cronbach’s α = 0.96, and the measure was found to be valid in terms of factorial, criterion, convergent, and discriminant validity (see Stoll-Kleemann et al., 2022b).

##### Social Desirability

To detect test falsification due to positive self-presentation and socially desirable response tendencies, the short version of social desirability scale (Satow, 2012) was used. The participants answered two items on a 4-point scale and thus the sum score ranged from 2 to 8. A value of 7 or higher indicated a high tendency to distort self-presentation and it is thus recommended to exclude participants scoring 7 or higher. Based on this criterion, we had to exclude 12 participants prior to the analysis. The final sample consisted of *N* = 174 participants, as described above.

**Sociodemographic variables** such as gender, age, education level, and working situation were queried directly.

#### Design and Procedure

We used *SoSci Survey* (Leiner, 2020) to prepare and host the online survey. The questionnaires analyzed in this article were part of a larger data collection comprising further questionnaires on moral constructs (see Stoll-Kleemann et al., 2022b). Starting with the sociodemographic variables, the questionnaires were answered in the following order: moral competence^1^, past pro-environmental behavior, moral disengagement in high carbon behavior, pro-environmental intention, moral disengagement in general^1^, idealism and relativism^1^, justice sensitivity, moral pride^1^, empathy and perspective taking^1^, moral identity^1^, and Machiavellianism^1^. Answering all questions took the participants between 10 and 34 min (*Mdn* = 19 min). The findings were considered significant in two-sided testing when *p* < 0.05. All analyses were conducted using *R* (R Core Team, 2020). In addition to established packages, the following were used: *bda* (Wang, 2021), *car* (Fox and Weisberg, 2019), *dplyr* (Wickham et al., 2022), *skedastic* (Farrar, 2020), and *lm.beta* (Behrendt, 2014).

### Results

To test the assumed positive prediction of the pro-social justice sensitivities and the assumed negative prediction of victim sensitivity and moral disengagement according to Hypotheses 1 and 2, we conducted two regression analyses with (a) pro-environmental intention and (b) past pro-environmental behavior as dependent variables, respectively. We included perpetrator, beneficiary, observer, and victim sensitivity as well as moral disengagement in high carbon behavior as predictors (Model A: on pro-environmental intention, Model B: on past pro-environmental behavior).

The prerequisites of no autocorrelation and normal distribution of residuals were given in both models. Model A showed multicollinearity, and Models A and B heteroscedasticity. As a linear model can be considered robust (Cohen, 2013), it was nevertheless used in this study. The regression tables of Models A and B are provided in Table 3.

**TABLE 3:** Regression analyses of pro-environmental intention and past pro-environmental behavior on justice sensitivity and moral disengagement in high carbon behavior in Study 1.

	Model A: Pro-environmental intention			Model B: Past behavior		
Effect	β	95% CI [LL, UL]	*p*	β	95% CI [LL, UL]	*p*
**Confirmatory analysis**						
Perpetrator sensitivity	0.02	[–0.12, 0.15]	0.793	0.08	[–0.07, 0.34]	0.295
Beneficiary sensitivity	0.04	[–0.09, 0.17]	0.532	–0.03	[–0.19, 0.13]	0.702
Observer sensitivity	**0.16**	**[**–**0.01, 0.31]**	**0.033**	0.16	[–0.01, 0.33]	0.058
Victim sensitivity	–0.08	[–0.22, 0.06]	0.266	–0.14	[–0.31, 0.02]	0.083
Moral disengagement	–**0.52**	**[**–**0.66,** –**0.39]**	** < 0.001**	–**0.33**	**[**–**0.49,** –**0.17]**	** < 0.001**
**Exploratory analysis**						
Perpetrator sensitivity	–0.03	[–0.13, 0.08]	0.468			
Beneficiary sensitivity	0.06	[–0.03, 0.16]	0.190			
Observer sensitivity	0.06	[–0.05, 0.16]	0.274			
Victim sensitivity	0.01	[–0.09, 0.11]	0.825			
Moral disengagement	–**0.31**	**[**–**0.41,** –**0.21]**	** < 0.001**			
Past behavior	**0.64**	**[0.55, 0.73]**	** < 0.001**			

*Model A: F(5, 168) = 20.08, p < 0.001, R^2^ = 0.37; Explorative Analysis: F(6, 167) = 66.78, p < 0.001, R^2^ = 0.71; Model B: F(5, 168) = 7.46, p < 0.001, R^2^ = 0.18. β = standardized regression weights. LL and UL indicate the lower and upper limits of a confidence interval of the standardized regression weights, respectively. Bold values indicate significant results.*

Model A significantly predicted pro-environmental intention, *F*(5, 168) = 20.08, *p* < 0.001, with a large effect size (according to Cohen, 2013), *R*^2^ = 0.37 (adjusted *R*^2^ = 0.36). Observer sensitivity (positive) and moral disengagement (negative) were significant predictors. All effects were in the hypothesized direction and, therefore, Hypotheses 1a and 2a were partially supported.

Model B predicted past pro-environmental behavior significantly, *F*(5, 168) = 7.46, *p* < 0.001, with *R*^2^ = 0.18 (adjusted *R*^2^ = 0.16), which is indicative of a medium effect size (Cohen, 2013). The only significant predictor was moral disengagement (negative). Except for beneficiary sensitivity, the effects were at least in the hypothesized direction, although they were not significant. As a result, Hypothesis 2b received only partial support.

Additionally, as past behavior was shown to be a strong predictor of pro-environmental intention (e.g., Bamberg et al., 2003; Carrus et al., 2008; Manca et al., 2020), we exploratively added past pro-environmental behavior in a second step in the regression analysis on pro-environmental intention. The standardized regression weights are shown in Table 3. Together, the predictors of this model (justice sensitivities, moral disengagement, past pro-environmental behavior) significantly predicted pro-environmental intention, *F*(6, 167) = 66.78, *p* < 0.001, with a large effect size (Cohen, 2013), *R*^2^ = 0.71 (adjusted *R*^2^ = 0.70). Adding past behavior as a predictor explained an additional 33% of the variance of pro-environmental intention, incremental *F*(1, 167) = 188.35, *p* < 0.001. While, perpetrator, beneficiary, and victim sensitivity remained non-significant predictors, moral disengagement in high carbon behavior was still significant. Observer sensitivity no longer significantly predicted pro-environmental intention. Past behavior was not only a significant, but also the strongest predictor in this model, followed closely by moral disengagement. Moreover, the inclusion of past behavior also reduced the explanatory power of moral disengagement.

In line with Hypothesis 5, moral disengagement in high carbon behavior was positively correlated to victim sensitivity, *r*(172) = 0.21, *p* = 0.005. As both variables were not normally distributed, Spearman’s rank correlation was additionally computed. This supported the result obtained with the Pearson correlation, ρ = 0.12, *p* < 0.001.

## Study 2

To replicate the findings of Study 1 (H1, 2, and 4) in a larger sample and to additionally test Hypotheses 3 and 5, we again measured the two dependent variables pro-environmental behavior and pro-environmental intention. However, to provide a potentially more accurate measure for environmentally friendly behavior, we used a carbon footprint calculator in Study 2. The independent variables again were the justice sensitivity perspectives and moral disengagement in high carbon behavior. We included moral emotions as predictors and guilt as a mediator.

### Materials and Methods

#### Participants

For the second study, we recruited 1,100 German participants in parallel to Study 1 (*via meinungsplatz.de*). Parallel quota ratios regarding the distribution of the German population for gender and age were set. We also applied the same exclusion criteria as in Study 1. Consequently, *n* = 121 participants were excluded due to the RSI (Leiner, 2019), and *n* = 99 due to potential socially desirable responses (Satow, 2012).

The final sample of Study 2 comprised 880 participants with 454 females and 426 males (age *M* = 50.43, *SD* = 17.0, range = 18–84 years). Two χ^2^-tests checked whether the age and gender groups of the reduced sample were still in line with the predefined quotas. We found no significant deviation from the proportions set for the recruiting procedure, either for gender, χ^2^(1) = 0.89, *p* = 0.345 or for age group, χ^2^(5) = 9.00, *p* = 0.109. Therefore, the final sample can still be regarded as representative of the German adult population in terms of age and gender (also see Table 4).

**TABLE 4:** Proportions of German age groups (in 2018) and the derived sampling goals along with the final sample proportions in Study 2.

			Sampling after exclusion		
Age group	Proportion in population	Quota sampling before exclusion	Female	Male	Total
18–25 years	12%	136	53	39	92
26–35 years	15%	166	65	55	120
36–45 years	14%	154	57	54	111
46–55 years	18%	200	87	82	169
56–65 years	16%	182	76	83	159
66 years or older	24%	262	116	113	229

*Proportions are based on Federal Statistical Office Germany (2019). We assumed gender to be equally distributed (50:50).*

A sensitivity analysis using G*Power (Faul et al., 2007) indicated that with the given sample size of *N* = 880, α = 0.05, and a power of 1 – β = 0.80, linear multiple regression with 10 predictors (four justice sensitivities, moral disengagement in high carbon behavior, guilt, shame, authentic and hubristic pride, gratitude) could detect small effect sizes of *R*^2^ = 0.02 and with a power of 1 – β = 0.95 could detect small effect sizes of *R*^2^ = 0.03. As these effect sizes are even smaller than those we found in Study 1, the sample size can be regarded as being sufficient to reliably quantify the effects.

#### Instruments

For pro-environmental intention, justice sensitivity, moral disengagement in high carbon behavior, and sociodemographic data, the same instruments from Study 1 were used. However, we exchanged the instrument for pro-environmental behavior and included scales for moral emotions. The descriptive statistics and internal consistencies of all variables of Study 2 can be found in Table 5.

**TABLE 5:** Descriptive statistics and internal consistency of all focal measures in Study 2.

Variable	*M*	*SD*	Skewness	Cronbach’s α
*Dependent variables*				
Pro-environmental behavior	11.12	8.04	14.81	−
Pro-environmental intention	4.20	0.67	–0.37	0.66
*Justice sensitivities*				
Perpetrator sensitivity	4.61	1.31	–0.90	0.87
Beneficiary sensitivity	3.00	1.34	0.17	0.88
Observer sensitivity	4.25	1.20	–0.69	0.79
Victim sensitivity	3.52	1.39	–0.14	0.81
Moral disengagement	2.40	1.01	0.58	0.95
*Moral emotions*				
Guilt proneness	3.52	1.50	–0.15	0.66
Shame proneness	3.60	1.52	–0.23	0.67
Authentic pride	4.52	1.17	–0.82	0.55
Hubristic pride	4.56	1.17	–0.82	0.54
Thankfulness	4.43	1.32	–0.72	0.78

*Cronbach’s α contains standardized internal consistencies.*

##### Pro-environmental Behavior (Carbon Footprint)

To obtain a more informative proxy measure of pro-environmental behavior, participants calculated their current individual carbon footprint with a carbon footprint calculator developed by the German Environment Agency (Umweltbundesamt [UBA], 2022). The calculator was embedded into the survey so that there was no need to leave the website. The questions covered the domains (1) living and electricity, (2) mobility, (3) diet, (4) other consumer behavior, and (5) public emissions. The participants had to complete forms asking for specific details of their daily life, e.g., personal annual mileage with one’s own car, via carsharing, bicycling, and public transport or whether they forgo the purchase of new products in favor of second-hand goods.

##### Pride, Guilt, and Shame-Proneness

In the lack of soundly validated *and* economic, i.e., brief, German scales to measure pride, guilt, and shame proneness, we designed four scenarios based on the Test of Self-Conscious Affect (TOSCA; Tangney et al., 2000) – two in which an intended pro-environmental behavior was (a) accomplished or (b) not accomplished, respectively. An example of such a scenario is the following: “For ecological reasons, you have decided to travel less by car and more by bicycle. Accordingly, you have planned to ride your bike the short distance to visit someone in the evening. However, when the time comes, you feel exhausted and take the car again.” Participants rated statements such as: “You would regret taking the car” (guilt-proneness), on a 6-point scale (1 = not likely, 6 = very likely). Thus, guilt, shame, and authentic and hubristic pride were each assessed with 2 items in 2 different scenarios, resulting in 8 items; and each scenario measured two emotions (shame and guilt, or authentic and hubristic pride). The original questionnaire was developed on the basis of descriptions of personal experiences of guilt, shame, and pride, and was found to successfully measure characteristic guilt- and shame-proneness as well as authentic and hubristic pride. The internal consistency was questionable for guilt (α = 0.66) and shame (α = 0.67) and poor for both dimensions of pride (α_authentic  pride_ = 0.55, α_hubristic  pride_ = 0.54). However, as the scales comprise only two items and these items were presented in the context of different scenarios which might have introduced a context variance, the reliabilities were regarded as acceptable and, therefore, sufficient for the planned analyses; the scenarios and items can be found in Appendix C.

##### Gratitude-Proneness in Climate Change

We asked the participants whether they are thankful for their privileges in the climate crisis (6-point scale; 1 = “totally disagree”, 6 = “totally agree”). The two items were: “I am grateful to live in a region of the world where climate change will arrive later and less extreme than in other regions” and “I am grateful to belong to a generation that is even less affected by climate change than future generations.” Cronbach’s α was acceptable (0.78). The items can also be found in Appendix C.

#### Design and Procedure

Again, we conducted an online survey with the help of *SoSci Survey* (Leiner, 2020). The time required to answer all questions ranged from 9.8 to 68.1 min, with 25.1 min being the median. The findings were considered significant in two-sided testing when *p* < 0.05. All analyses were conducted using *R* (R Core Team, 2020) using the same packages from Study 1.

### Results

According to Hypotheses 1 and 2, we again assumed a positive prediction of the pro-social justice sensitivities and a negative prediction of the pro-self sensitivity and moral disengagement. Additionally, we investigated Hypothesis 3 which stated that there is an association between moral emotions and pro-environmental behavior and intention. All three hypotheses were tested by calculating multiple regression analyses on (a) pro-environmental intention and (b) pro-environmental behavior. The prerequisites of no autocorrelation and multicollinearity were given for both regression models on pro-environmental behavior and pro-environmental intention. Homoskedasticity was given for Model B (pro-environmental behavior), but not for Model A (pro-environmental intention), and normal distribution of residuals was not given in both models. As a regression analysis can be considered robust (Cohen, 2013), it was nevertheless used in this study.

When testing Hypotheses 1a, 2a, and 3a, the model comprising justice sensitivities, moral emotions, and moral disengagement significantly predicted pro-environmental intention (Model A), *F*(10, 869) = 38.27, *p* < 0.001, *R*^2^ = 0.31 (adjusted *R*^2^ = 0.30), whereby significant predictors were perpetrator and beneficiary sensitivity, moral disengagement, and authentic pride. However, observer and victim sensitivity, guilt, shame, hubristic pride, and gratitude were non-significant (see Table 6 for regression weights).

**TABLE 6:** Regression analyses of pro-environmental intention and behavior *via* justice sensitivity, moral disengagement in high carbon behavior, and moral emotion in Study 2.

	Model A: Pro-environmental intention			Model B: Pro-environmental behavior		
Effect	β	95% CI [LL, UL]	*p*	β	95% CI [LL, UL]	*p*
Perpetrator sensitivity	**0.12**	**[0.06, 0.19]**	** < 0.001**	0.06	[–0.02, 0.14]	0.145
Beneficiary sensitivity	–**0.08**	**[**–**0.15,** –**0.01]**	**0.018**	–**0.08**	**[**–**0.16, 0.16]**	**0.050**
Observer sensitivity	0.06	[–0.01, 0.13]	0.100	0.02	[–0.07, 0.10]	0.725
Victim sensitivity	–0.02	[–0.08, 0.04]	0.576	< 0.01	[–0.07, 0.07]	0.998
Moral disengagement	–**0.30**	**[**-**0.37,** –**0.23]**	** < 0.001**	**0.82**	**[0.02, 0.18]**	**0.011**
Guilt	0.06	[–0.05, 0.16]	0.301	0.01	[–0.11, 0.14]	0.830
Shame	0.04	[–0.07, 0.14]	0.498	–0.03	[–0.16, 0.10]	0.643
Authentic Pride	**0.14**	**[0.04, 0.24]**	**0.007**	–0.03	[–0.15, 0.10]	0.677
Hubristic Pride	0.09	[–0.01, 0.02]	0.079	0.05	[–0.08, 0.17]	0.462
Gratitude	–0.03	[–0.09, 0.02]	0.253	< 0.01	[–0.07, 0.07]	0.993

*Model A: F(10, 869) = 38.27, p < 0.001, R^2^ = 0.30; Model B: F(10, 869) = 1.36, p = 0.197, R^2^ = 0.02. β = standardized regression weights. LL and UL indicate the lower and upper limits of a confidence interval of the standardized regression weights, respectively. In Study 2, higher scores in pro-environmental behavior indicate a higher carbon footprint. Predictors should be interpreted in line with this. Bold values indicate significant results.*

A multiple regression model comprising the justice sensitivities, moral emotions, and moral disengagement in high carbon behavior failed to significantly predict pro-environmental behavior (Model B), *F*(10, 869) = 1.36, *p* = 0.197. Table 6 shows the regression weights of all predictors. Contrary to expectations, moral disengagement was shown to be the only significant predictor whereas perpetrator, beneficiary, observer, and victim sensitivity, guilt, shame, authentic and hubristic pride, and gratitude were non-significant predictors. Hence, Hypotheses 1b, 2b, and 3b were not supported.

**FIGURE 2:**
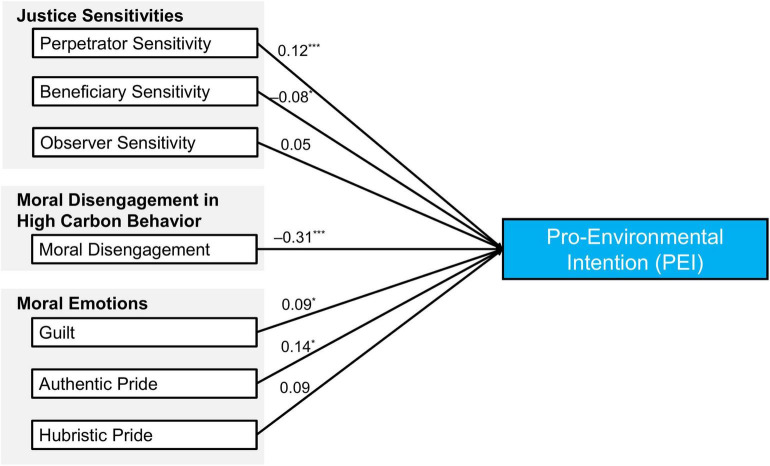
Regression weights of the final model of pro-environmental intention after backward elimination. This final model significantly predicted pro-environmental intention, *F*(7, 872) = 54.41, *p* < 0.001, with a large effect size, *R*^2^ = 0.30 (adjusted *R*^2^ = 0.29). *p < 0.05, ***p < 0.001; coefficients are standardized regression weights.

To reduce our comparably complex model on pro-environmental intention, a stepwise regression analysis with statistical backward elimination (based on AIC) was conducted to identify the relevant predictors. According to a backward-elimination strategy, victim sensitivity, shame proneness, and gratitude were excluded from the final regression model (*p* > 0.252). This final model (Model A-) can be found in Table 7 as well as in Figure 2. Consequently, the final model included the following predictors: perpetrator, beneficiary, and observer sensitivity, moral disengagement in high carbon behavior, guilt-proneness, and authentic and hubristic pride. This final model significantly predicted pro-environmental intention, *F*(7, 872) = 54.41, *p* < 0.001, with a large effect size, *R*^2^ = 0.30 (adjusted *R*^2^ = 0.29).

**TABLE 7:** Regression model of pro-environmental intention after backward elimination of Model A.

	Model A		
Effect	β	95% CI [LL, UL]	*p*
Perpetrator sensitivity	**0.12**	**[0.06, 0.19]**	** < 0.001**
Beneficiary sensitivity	–**0.08**	**[**–**0.15,** –**0.01]**	**0.017**
Observer sensitivity	0.05	[–0.01, 0.12]	0.121
Moral disengagement	–**0.31**	**[**–**0.37,** –**0.24]**	** < 0.001**
Guilt	**0.09**	**[0.02, 0.15]**	**0.013**
Authentic pride	**0.14**	**[0.03, 0.24]**	**0.010**
Hubristic pride	0.09	[–0.01, 0.19]	0.082

*F(7, 872) = 54.41, p < 0.001, R^2^ = 0.30. β = standardized regression weights. LL and UL indicate the lower and upper limits of a confidence interval of the standardized regression weights, respectively. Bold values indicate significant results.*

**FIGURE 3:**
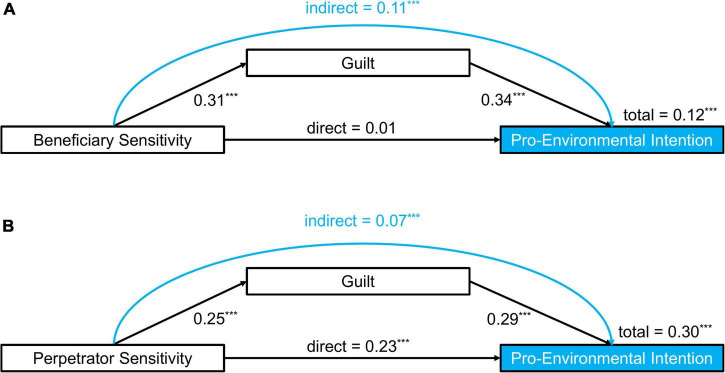
Mediation analyses of guilt on the effect of **(A)** Beneficiary sensitivity [respectively **(B)** Perpetrator sensitivity] on pro-environmental intention. ^***^p < 0.001; coefficients are standardized regression weights.

A mediation analysis in line with Baron and Kenny (1986) was performed to test Hypothesis 4a, i.e., whether the prediction of beneficiary sensitivity for pro-environmental intention would be mediated by guilt-proneness. In step 1 of the mediation model, the regression of pro-environmental intention on beneficiary sensitivity, ignoring the mediator, was significant, β = 0.12, *p* < 0.001. Step 2 showed that the regression of the mediator (guilt) on beneficiary sensitivity was also significant, β = 0.31, *p* < 0.001. Step 3 of the mediation analysis showed that the mediator (guilt), controlling for beneficiary sensitivity, significantly predicted pro-environmental intention, β = 0.34, *p* < 0.001. Step 4 of the analysis revealed that controlling for the mediator (guilt), beneficiary sensitivity was not a significant predictor of pro-environmental intention, β = 0.01, *p* = 0.708. According to a Sobel test (*z* = 7.06, *p* < 0.001), guilt fully mediated the relationship between beneficiary sensitivity and pro-environmental intention. This mediation is illustrated in Figure 3.

A second mediation with the same procedure (Baron and Kenny, 1986) was performed to analyze whether perpetrator sensitivity predicts pro-environmental intention and whether the direct path would be mediated by guilt (Hypothesis 4b). In step 1 of the mediation model, the regression of pro-environmental intention on perpetrator sensitivity, ignoring the mediator, was significant, β = 0.30, *p* < 0.001. Step 2 showed that the regression of the mediator (guilt) on perpetrator sensitivity was also significant, β = 0.25, *p* < 0.001. Step 3 of the mediation analysis showed that the mediator (guilt), controlling for perpetrator sensitivity, significantly predicted pro-environmental intention, β = 0.29, *p* < 0.001. Step 4 of the analysis revealed that controlling for the mediator (guilt), the predictive power of beneficiary sensitivity on pro-environmental intention was reduced, β = 0.23, *p* ≤ 0.001. Again, a Sobel Test showed that this mediation is significant (*z* = 5.81, *p* < 0.001). Therefore, guilt partially mediated the relationship between perpetrator sensitivity and pro-environmental intention. The second mediation analysis is also illustrated in Figure 3.

Confirming Hypothesis 5, victim sensitivity was again positively and significantly correlated with moral disengagement in high carbon behavior, *r*(878) = 0.13, *p* < 0.001. As both variables were not normally distributed, Spearman’s rank correlation was again computed which returned the same result, ρ = 0.14, *p* < 0.001.

## Discussion

The aim of this study was to investigate the role of justice sensitivity, moral disengagement, and moral emotions on pro-environmental intention and behavior. In Study 1, moral disengagement in high carbon behavior was able to significantly and negatively predict past pro-environmental behavior, while the four justice sensitivities did not. A pro-environmental intention was significantly predicted by moral disengagement in high carbon behavior (negatively) and observer sensitivity (positively). Therefore, Hypotheses 1 and 2 were only partially supported. An attempt to replicate these results in a larger sample (Study 2) with the exact carbon footprint calculator as a proxy for pro-environmental behavior failed. However, the pro-environmental *intention* was significantly predicted in the larger sample by justice sensitivities, moral disengagement, and moral emotions. In the final model, after a backward elimination process, perpetrator sensitivity, guilt, and hubristic pride emerged as significant positive predictors and beneficiary sensitivity and moral disengagement as significant negative predictors. Victim sensitivity, shame, and gratitude were excluded because their explanatory power was too low compared to the other predictors. Consequently, Hypothesis 3 was only partially supported. Based on the results, lower moral disengagement served as the best predictor for (more) pro-environmental intention and behavior in both studies. However, an exploratory analysis in Study 1 showed that past behavior was an even stronger predictor of pro-environmental intention. Whereas beneficiary sensitivity was a significant negative predictor in the final model on pro-environmental intention (Model B), its direct effect on pro-environmental intention in the mediation analysis was positive, as hypothesized. In line with Hypothesis 4, the predictions of perpetrator and beneficiary sensitivity were mediated by guilt, which is the emotion that occurs at high levels in each of these two justice sensitivities.

In the light of these findings and the cognitive appraisal theory of emotions (Arnold, 1960), we support Baumert et al. (2013b) explanation that the perception of injustice (here: beneficiary and perpetrator sensitivity) elicits moral emotions (here: guilt), which in turn lead to an action tendency (here: pro-environmental intention). This theoretical model should be considered for further research on the role of justice sensitivity and moral emotions on pro-environmental intention and may be included in models of pro-environmental behavior.

Consistent with Hypothesis 5, victim sensitivity was positively related to moral disengagement, which is a strong negative predictor of pro-environmental behavior and intention in our study. Individuals with high victim sensitivity thus are more prone to morally disengage and justify their actions instead of taking responsibility. As we outlined in the Introduction, pro-environmental behavior requires accepting responsibility. We regard taking responsibility and moral disengagement as opposites, especially in the area of pro-environmental intention and behavior. Detert et al. (2008) found factors that inhibit or facilitate moral disengagement. While empathy, perspective-taking, moral identity, female gender, and an internal locus of control were found to be inhibitors of moral disengagement, trait cynicism and an external locus of control were found to facilitate moral disengagement. According to our findings, victim sensitivity can be added to the list of facilitators. Therefore, people with high victim sensitivity, who rather tend to morally disengage than to take responsibility are the brakemen of socio-ecological transformation, as they are not willing to act for the sake of the common good but only for their own benefit. The twisted part is that these people see themselves as victims. They argue, for example, that something is taken away from them or that they are disadvantaged. This can be observed in various social debates as the following two examples show: First, in Germany, members of the right-wing populist party Alternative für Deutschland (AfD) have been portraying themselves as victims of political correctness for years as a political strategy (Heinze, 2021). Second, deniers of the COVID-19 pandemic reinforce this perception by wearing a Jewish badge (historic symbol of discrimination, originally used to discriminate Jewish people in Nazi-Germany) with the inscription “unvaccinated” (Fröhlich, 2022). What is striking here is the general pattern of using self-staging as a victim as a political strategy in radical right-wing movements (Weiß, 2011), which in turn increasingly attracts victim sensitives. A current study by Jahnke et al. (2020) provides empirical evidence that victim sensitivity predicted stronger right-wing orientations as well as general and right-wing radicalization.

Although not all justice sensitivities significantly predicted pro-environmental behavior and pro-environmental intention in all models, most of our hypotheses were supported. The considered constructs performed better at predicting pro-environmental intention than behavior and past behavior. This could be due to the intention-behavior gap, which describes that intended behavior is not always implemented (Sheeran, 2002). It is conceivable that the constructs used here refer only to intention, but not to implementation. Also, we may have included too many and too similar predictors. Although multicollinearity was absent (except for Model A in Study 1), perpetrator, beneficiary, and observer sensitivity showed high concordance in previous studies (Münscher, 2017). Therefore, some authors (e.g., Jahnke et al., 2020) use only one pro-social justice sensitivity. Another approach could be to aggregate these measures (e.g., by means of factor scores) into an umbrella score for pro-social sensitivities. We further assume that the high predictive power of moral disengagement undermines the prediction of the justice sensitivities, especially the similar effect of victim sensitivity. However, moral disengagement in high carbon behavior and victim sensitivity were correlated but not as high as they would reflect an overarching construct. Although any association with pro-environmental *behavior* was weak in both studies (which also means in the two different scales that were used), we found that victim sensitivity and moral disengagement negatively predicted pro-environmental *intention*, while perpetrator, beneficiary, and observer sensitivity positively predicted pro-environmental intention. This may be explained by different degrees of responsibility taking: Those who perceive injustice from the perspective of the beneficiary, observer, or perpetrator are more likely to feel responsible (Ehrhardt-Madapathi et al., 2018). A greater sense of responsibility, in turn, is a promising predictor to engage in pro-environmental behavior (Kollmuss and Agyeman, 2002).

Given these diametrical effects of pro-social and pro-self sensitivities, it seems promising to investigate whether the pro-social perspectives of justice sensitivity can be trained and increased. A preliminary study in this area (Maltese et al., 2013) shows that specific training can teach people to recognize the consequences of their behavior as being unjust in ambiguous situations. In a subsequent game, this classification led people to use more of their own resources to restore justice, in contrast to the control group. Applied to the context of the climate crisis, the aim would be to draw attention to why the climate crisis is a matter of justice and how one’s own behavior is connected to the unjust consequences of the climate crisis. Another example is the intervention of Malan et al. (2020) who were able to motivate university students to reduce their high carbon-emitting meat consumption by highlighting the impacts on social justice (and environmental sustainability).

One may argue that a focus on the consequences of individual behavior may lead to reactance, as the resulting emotion of perpetrator and beneficiary sensitivity is guilt, and people seek to protect themselves from negative emotions. Indeed, in our study, moral disengagement in high carbon behavior had remarkable predictive power. According to the theory of moral disengagement (Bandura, 2016), emotional distress (an aversive negative affective state) is unpleasant to endure. As a result, individuals rather change their argumentation (via justifications) than their behavior. Therefore, a focus on negative emotions may lead to increasing moral disengagement instead of a behavioral change. That is why Chapman et al. (2017) recommended that a nuanced and authentic approach is necessary when emotions are used to target pro-environmental behavior change. However, we state that outlining means of behavior change, rather than just pointing out what is being done wrong, induces anticipated pride. Both authentic and hubristic pride withstood a backward elimination as predictors of pro-environmental intention, and, therefore, should be considered in attempts to change behavior. This is also in line with the literature on the positive effects of pride on pro-environmental behavior (Bissing-Olson et al., 2016). Notwithstanding, the effect of pride is not consistent but rather depends on the context (Hurst and Sintov, 2022), which again supports the necessity of careful use of emotions in pro-environmental behavior change.

However, another positive emotion relevant for pro-environmental behavior and intention which we included in our study, namely gratitude, was undermined by the backward elimination procedure and thus its effect on pro-environmental behavior can be assumed to be small in comparison to justice sensitivity, moral disengagement, guilt, and pride. As we outlined in the Introduction, we expected gratitude for privileges to be a generalized emotion shaping behavior (Landmann, 2020). Currently, there is not much literature on the role of gratitude in pro-environmental behavior. Yet, this finding potentially challenges a recent finding on the positive effect of gratitude on pro-environmental intention (Tam, 2022). This may be rooted in Tam’s concept of gratitude, which can also be regarded as a trait but directly refers to gratitude toward nature rather than one’s own privileges in the face of climate injustice. Gratitude toward nature is similar to and associated with connectedness to nature, which also is a positive predictor of pro-environmental behavior (Kals and Maes, 2002; Tam, 2022). Contrary to gratitude toward nature, gratitude toward one’s own privileges requires a high level of reflectiveness as one has to not take comfortable benefits for granted (e.g., mobile devices whose batteries are manufactured under exploitative working conditions and water pollution; Wanger, 2011).

This ability to recognize one’s own privileges, however, may also lead to existential guilt instead of gratitude toward privileges. Existential guilt describes the moral emotion of a person who benefits from illegitimate privileges and occurs in individuals who causally link their own privileges to others’ deprivation, and can see their advantages as the results of a circumstance that they are able to control (Montada et al., 1986). It mainly arises in individuals with a high beneficiary sensitivity (Gollwitzer et al., 2005). In the current study, guilt proneness was positively predicted by both, beneficiary and perpetrator sensitivity.

The positive prediction of pro-environmental intention by guilt stands in line with existing literature (Carrus et al., 2008; Rees et al., 2014; Hurst and Sintov, 2022). However, in our study, guilt was not only a significant predictor but also a mediator. We, therefore, recommend devoting more attention to different theoretical and statistical associations than simple predictions. The data of our study suggest that the predictive power of shame was comparably small which is consistent with the results of an experimental study by Rees et al. (2014) who showed that the combination of guilt and shame into a “guilty conscience” construct was a stronger predictor of pro-environmental behavior than each of them taken separately. However, in their study, it was shame and not guilt that transformed the intention into manifest behavior. Consequently, shame may play a role in the implementation of pro-environmental intentions. Yet, the body of literature on shame is not as abundant as is on guilt; therefore, it is difficult to compare their respective influences.

The approach of combining emotions, as Rees et al. (2014) presented, nevertheless seems to be promising. When considering emotions, it is important to note that they rarely occur in isolation, but often as a blend. A current qualitative study (Marczak et al., 2022) shows that the emotional landscape concerning climate change is not limited to categories like, e.g., “anger,” but rather includes various specific emotions such as exasperation, irritation, frustration, impatience, annoyance, disgust, anger, and rage, as these are related to a perceived lack of commitment to climate action (these are only the emotions in the area of “anger”). A quantitative study (Stanley et al., 2021) confirmed that feeling each of three common climate emotions, fear, anger, and sadness, was a strong predictor of also experiencing the other two and hence the role of emotions under realistic conditions and not just in isolation from other emotions should be investigated. In line with this, Chapman et al. (2017) criticized the current research for overemphasizing the role of single emotions and, as a result, more studies investigated emotional profiles instead of single emotions in the context of the environment. Fernando et al. (2014) found, e.g., that sympathy for specific groups of people alone hardly produces intentions to act, but instead requires a (pro-social) emotional profile of sufficient sympathy, shame, and anger at the government and one’s ingroup, as well as some anger about those affected and some pride. Another example is the study of Wang et al. (2018), who found an emotional profile that was associated with high acceptance of environmental protection measures. This profile consisted of higher values for anger, anxiety, guilt, shame, powerlessness, and desperation (called the self-blame profile). Therefore, addressing emotions as patterns rather than single individual phenomena seems promising to consider in further investigations on pro-environmental behavior and intention.

Given the predictive power of guilt, and authentic pride, we agree with Landmann (2020) that addressing (already existing) generalized emotions may be more effective than single (induced) emotional episodes. We assume the proneness or dispositional tendency to experience a specific type of emotion to play a key role in motivating pro-environmental behavior. Being used in such a way, emotion research can help to better understand the psychological processes in pro-environmental intention and behavior. Roeser (2012) confirms that emotions may help to see something that purely rational assessments fail to discover. In her study, information about climate change with emotional content provided better insight into its moral meaning, while also providing a deeper, more reliable source of motivation for action than information without emotional content. Consequently, especially in the domain of the psychology of justice and morality, emotions are central and should receive more attention from environmental psychologists.

### Limitations

This study has some limitations concerning (1) the design, (2) the instruments, and (3) the sample. First of all, we used a correlational design which is not suitable for conclusions on causal relations between variables. Further research may expand our study design and derive more suitable experimental designs. Additionally, we only gave one possible order of the questionnaires involved in this study. However, having previously presented a scale such as moral disengagement in high carbon behavior might have influenced the subsequent scales such as pro-environmental intention. Since moral disengagement is used as a justification for not behaving in a pro-environmental manner, responses on the pro-environmental intention scale might have been lower than if the moral disengagement scale had been administered subsequently. However, we wanted pro-environmental intention to be answered not directly after pro-environmental behavior as we hypothesized this would cause the participants to repeat their answers from pro-environmental behavior instead of actually elaborating on their pro-environmental intention. Furthermore, some other emotions that are associated with justice sensitivity (e.g., anger, or moral outrage) could be included in further investigations. Also, as mentioned before, qualitative approaches seem promising to get a broader insight into the emotional landscape in the context of climate justice. Second, questionnaires are based on self-reported behavior, which may introduce biased responses. To counteract this, we followed the recommendations of Satow (2012) and used a measure to exclude the data from socially desirable responding individuals. Due to the lack of an appropriate measure, we established a measure of the proneness to various emotions in the climate context for this study (see Methods). However, this measure was not validated. Also, the use of the carbon footprint calculator from Study 2 requires a lot of time and asks for very detailed information which may frustrate participants and lead them to not fill in the questionnaire conscientiously or even refrain from completing it at all. Additionally, standard answers are set in the carbon footprint calculator which may influence the answers of the participants. Third, our objective was to acquire a representative sample which is why we aimed to get a large sample size in Study 2. However, considering the high power of our calculations, we may have “oversampled” this second sample. A sample size that is that large may lead to an increased Type I error. At the same time, effect sizes, which we also used, are smaller in larger samples and account for this. Additionally, as we used the platform *meinungsplatz.de* to recruit panelists, the participants may be more affine toward digital media than other social groups as they created an account on that platform.

## Conclusion

We showed that pro-social justice sensitivities are able to (at least partially) predict pro-environmental behavior and intention. In our study, guilt mediated the relationship between justice sensitivity and pro-environmental intention. Authentic pride was also found to be a significant predictor of pro-environmental intention. Moral disengagement in high carbon behavior turned out to be a barrier to pro-environmental behavior change and is associated with dispositional victim sensitivity.

Consequently, we recommend rendering the aspects of justice, e.g., the consequences for the most vulnerable people, in climate change more saliently—both in future research interventions and climate communication by political agents or the media. The current Intergovernmental Panel on Climate Change [IPCC] (2022) started off strongly by consistently demanding more equitable societies. With great confidence, the authors thus declare that “solutions based on equity and social and climate justice reduce risks and enable climate resilient development” (p. 31). Climate justice is not only the ultimate goal of pro-environmental behavior, but it can also function as its elicitor by being a fundamental human motive that triggers strong moral emotions.

However, the realization of being a beneficiary of an unjust *status quo* can also be challenging and may elicit negative emotions. These negative emotions can lead to moral disengagement, especially when individuals score high in victim sensitivity. Therefore, the challenge when highlighting privilege is to ensure that the target group is supported when feeling negative emotions so that it does not slip into moral disengagement. For people who are already highly victim-sensitive, specific strategies should be designed that can liberate them from their suspicion and fear of experiencing a disadvantage.

Making the injustice of climate change more salient may thus be the first step toward achieving more climate-just societies.

## Data Availability Statement

The raw data supporting the conclusions of this article will be made available by the authors, without undue reservation.

## Ethics Statement

Both studies were designed and conducted in accordance with the Code of Ethics of the World Medical Association (2013) (“World Medical Association Declaration of Helsinki”). The participants in the two studies gave their informed consent to participate prior to the start of the survey. Ethical review and approval were not required for these studies in accordance with the German legislation and with institutional requirements. The consent of the data protection officer of the University of Greifswald has been obtained. The patients/participants provided their written informed consent to participate in this study.

## Author Contributions

SN conceived of the presented idea and wrote the first draft. SN, PF, and SS-K contributed to conception and design of the study. PF organized the database. SN and PF performed the statistical analysis. All authors contributed to manuscript revision, read, and approved the submitted version.

## Conflict of Interest

The authors declare that the research was conducted in the absence of any commercial or financial relationships that could be construed as a potential conflict of interest.

## Publisher’s Note

All claims expressed in this article are solely those of the authors and do not necessarily represent those of their affiliated organizations, or those of the publisher, the editors and the reviewers. Any product that may be evaluated in this article, or claim that may be made by its manufacturer, is not guaranteed or endorsed by the publisher.

## Footnotes

1 These questionnaires are not the subject of this article.

## Appendix

### Appendix A

Questionnaire on past pro-environmental behavior. Participants answered “yes,” “no,” or “do not know.” (R) indicates inversed items.

To what extent do these statements apply to you?

– In 2019, I mainly used a car for short distances (up to 20 km). (R)
– In 2019, I took trips by plane. (R)
– In 2019, I ate a vegetarian diet.
– In 2019, I purchased green electricity.
– In 2019, I switched off my electrical appliances completely (i.e., not only put them on standby).
– In 2019, I washed my laundry mainly at 60°C or higher. (R)
– In 2019, I paid more attention to the long term than to the price when making new purchases (e.g., electrical appliances, clothing).
– In 2019, I bought regional products rather than products transported over long distances.

– In 2019, I bought mainly waste-avoiding products (e.g., using no plastic bags for fruit and vegetables).
– In 2019, I heated my apartment to more than 22°C in the winter months. (R)

*Note.* To achieve a higher internal consistency, we excluded items with an (X), as described in the Methods section.

### Appendix B

Questionnaire on pro-environmental intention. Participants answered on a 6-point-scale from “does not apply at all” to “fully applies”. (R) indicates inversed items.

To what extent do these statements apply to you?

– In 2021, I would like to (continue to) mainly use a car for short distances (up to 20 km). (R)
– In 2021, I would like to, continue to) take trips by plane. (R)
– In 2021, I would like to (continue to) eat a vegetarian diet.
– In 2021, I would like to (continue to) purchase green electricity.
– In 2021, I would like to (continue to) switch off my electrical appliances completely (i.e., not only put them on standby).
– In 2021, I would like to (continue to) wash my laundry mainly at 60°C or higher. (R)
– In 2021, I would like to (continue to) pay more attention to the long term than to the price when making new purchases (e.g., electrical appliances, clothing).
– In 2021, I would like to (continue to) buy regional products rather than products transported over long distances.
– In 2021, I would like to (continue to) buy mainly waste-avoiding products (e.g., using no plastic bags for fruit and vegetables).
– In 2021, I would like to (continue to) heat my apartment to more than 22°C in the winter months. (R)

### Appendix C

Questionnaire on moral emotions (guilt, shame, authentic pride, hubristic pride, thankfulness) in pro-environmental behavior. Participants answered on a 6-point-scale for each scenario, ranging from “do not agree at all” to “strongly agree”.

Now we would like to ask you about the degree to which you agree with some statements regarding various imaginary scenarios. Please try to identify with these scenarios and imagine how you would act in this type of situation.

*1) For ecological reasons, you have decided to travel less by car and more by bicycle. Accordingly, you have planned to ride your bike the short distance to visit someone in the evening. However, when the time comes, you feel exhausted and take the car again.*

To what extent do you agree with the following statements?

– You would feel uncomfortable
– You would regret taking the car

*2) Because the carbon footprint of meat consumption is so high, you want to eat less meat, but this is generally difficult for you. However, because you came across some new vegetarian grilling recipes you found it easy not to eat meat at the last barbecue.*

To what extent do you agree with the following statements?

– You would think: “I can do more than I thought I could.”
– You would think: “I did a good job.”

*3) Due to the poor carbon dioxide balance, you would like to fly less. However, you would like to visit a somewhat distant destination, and because it takes twice as long to get there by train as by plane, you book a last-minute flight.*

To what extent do you agree with the following statements?

– You would feel uncomfortable about being inconsistent.
– You would regret not acting according to your principles.

*4) For the sake of the environment, you have resolved to save electricity. However, you have a lot of devices you don’t want to unplug every evening, so you buy a multi-socket device with a toggle switch. This means the devices don’t just go into standby mode, but are actually switched off.*

To what extent do you agree with the following statements?

– You would be happy to have found a solution yourself.
– You would think that you have handled the situation well.

*5) To what extent do you agree with the following general statements (without reference to the scenarios)?*

– I am grateful to live in a region of the world in which climate change will arrive later and be less extreme than in other regions.
– I am grateful to live at a time less affected by climate change than future generations apparently will be.
